# Obinutuzumab plus fludarabine and cyclophosphamide in previously untreated, fit patients with chronic lymphocytic leukemia: a subgroup analysis of the GREEN study

**DOI:** 10.1038/s41375-019-0554-1

**Published:** 2019-08-27

**Authors:** Francesc Bosch, Guy Cantin, Agostino Cortelezzi, Wolfgang Knauf, Mourad Tiab, Mehmet Turgut, Andrey Zaritskey, Jean-Louis Merot, Eugen Tausch, Kerstin Trunzer, Susan Robson, Ekaterina Gresko, Sebastian Böttcher, Robin Foà, Stephan Stilgenbauer, Véronique Leblond

**Affiliations:** 1 University Hospital Vall d’Hebron, Barcelona, Spain; 2 Hopital De L’Enfant—Jesus, Quebec City, QC Canada; 3 Hematology Unit, Policlinico Hospital and University of Milan, Milan, Italy; 4 Onkologische Gemeinschaftspraxis, Agaplesion Bethanien Krankenhaus, Frankfurt, Germany; 5 University Hospital, La Roche Sur Yon, France; 6 Ondokuz Mayis University, Samsun, Turkey; 7 Almazov National Medical Research Center, St Petersburg, Russia; 8 IQVIA, St Ouen, France; 9 Department of Internal Medicine III, Ulm University, Ulm and Innere Medizin I, Universitätsklinikum des Saarlandes, Homburg, Germany; 10 F. Hoffmann-La Roche Ltd, Basel, Switzerland; 11 Second Department of Medicine, University of Schleswig-Holstein, Campus Kiel, Kiel, Germany and Clinic III, Hematology, Oncology and Palliative Medicine, Rostock University Medical Center, Rostock, Germany; 12 Hematology, Sapienza University, Rome, Italy; 13 Sorbonne Université, AP-HP Hôpital Pitié Salpêtrière, Paris, France

**Keywords:** Haematological cancer, Prognosis

## Abstract

GREEN (NCT01905943) is a nonrandomized, open-label, single-arm, phase 3b study investigating the safety and efficacy of obinutuzumab alone or in combination with chemotherapy in chronic lymphocytic leukemia (CLL). We report the preplanned subgroup analysis of 140 previously untreated, fit CLL patients who received obinutuzumab plus fludarabine and cyclophosphamide (G-FC). The primary endpoint was safety and tolerability. Efficacy was the secondary endpoint. Obinutuzumab 1000 mg was administered intravenously on Day (D)1 (dose split D1‒2), D8 and D15 of Cycle (C)1, and D1 of C2–6 (28-day cycles). Standard intravenous/oral doses of fludarabine and cyclophosphamide were administered on D1–3 of C1–6. Overall, 87.1% of patients experienced grade ≥ 3 adverse events (AEs), including neutropenia (67.1%) and thrombocytopenia (17.1%). Serious AEs were experienced by 42.1% of patients. Rates of grade ≥ 3 infusion-related reactions and infections were 19.3% and 15.7%, respectively. Overall response rate was observed in 90.0%, with 46.4% of patients achieving complete response (CR; including CR with incomplete marrow recovery). Minimal residual disease negativity rates were 64.3% in peripheral blood and 35.7% in bone marrow (intent-to-treat analysis). After a median observation time of 25.6 months, 2 year progression-free survival was 91%. Frontline G-FC represents a promising treatment option for fit patients with CLL.

## Introduction

Chronic lymphocytic leukemia (CLL) is the most common leukemia in Western countries, with an annual, age-adjusted incidence of approximately four per 100,000 inhabitants [1, 2]. Frontline treatment decisions in symptomatic patients are generally based on age, physical fitness, and the presence of genetic risk factors, such as chromosome 17p deletion and *TP53* gene mutation [1–4]. Although the therapeutic landscape in CLL has experienced a dramatic shift in recent years with the advent of new biologic agents, e.g., ibrutinib [5–10], chemoimmunotherapy remains a well-established frontline approach for a subset of patients with CLL [1, 11–19].

The combination of the anti-CD20 antibody, rituximab, plus fludarabine, and cyclophosphamide (R-FC) has been considered the standard of care for previously untreated, young, fit CLL patients who are eligible for this chemoimmunotherapy [16, 20]. This regimen significantly improves progression-free survival (PFS) and overall survival (OS) compared with FC alone [11, 20–23], and provides a superior PFS benefit compared with rituximab plus bendamustine [12]. Higher rates of minimal residual disease (MRD) negativity are also achieved with R-FC compared with FC alone [24], and there is growing evidence that therapies capable of eliminating MRD lead to improved clinical outcome [24–31]. Although R-FC currently remains the standard of care, the therapeutic paradigm is constantly evolving. Recent interim analysis of ibrutinib in combination with rituximab (IR) has demonstrated superior efficacy and a favorable safety profile versus R-FC [32]. However, while a subset of CLL patients receiving R-FC or IR as frontline therapy experience durable remission rates [11, 23, 32–36], the majority of patients are still destined to relapse. Obinutuzumab (GA101) is a glycoengineered type II anti-CD20 antibody that was developed in an attempt to improve therapeutic efficacy compared with rituximab. To date, obinutuzumab has demonstrated substantial activity in CLL [14, 17, 18, 37–39], and the additional benefit of obinutuzumab-based chemoimmunotherapy over rituximab-based chemoimmunotherapy has been observed in the pivotal phase 3 CLL11 trial [14, 18, 19]. In the CLL11 trial, obinutuzumab plus chlorambucil (G-Clb) provided a significant prolongation of PFS and OS in patients with CLL and comorbidities compared with rituximab plus chlorambucil (R-Clb) [14, 18, 19]. G-Clb also had an acceptable toxicity profile. While an increased risk of infusion-related reactions (IRRs) and neutropenia was observed, these adverse events (AEs) were manageable [14].

This superior efficacy of G-Clb over R-Clb, as seen in CLL11, has led to interest in evaluating the clinical activity of obinutuzumab in combination with other chemotherapies, such as bendamustine and FC [39, 40]. To date, frontline obinutuzumab plus bendamustine (G-B) has shown encouraging efficacy and manageable toxicity in patients with CLL [40]. Data from the phase 1b feasibility study (GALTON) have shown that frontline G-FC is also tolerable and shows signs of clinical activity in CLL [39], but larger, confirmatory studies are needed.

GREEN (NCT01905943) is a phase 3b safety study of obinutuzumab alone or in combination with chemotherapy (investigator’s choice) in 972 patients with previously untreated or relapsed/refractory CLL, including both fit and unfit patients. This paper reports the safety and efficacy results from a pre-specified subgroup of 140 previously untreated, fit patients who received G-FC in GREEN.

## Subjects and methods

### Study design and patients

GREEN is an international, multicenter phase 3b safety study, with a nonrandomized, noncomparative, open-label design. The primary objective is to assess the safety and tolerability of obinutuzumab alone or in combination with various chemotherapy regimens. Chemotherapy options were partly dependent on patient fitness and based on investigator’s choice. Assessment of efficacy is the secondary objective. In an attempt to mitigate the risk of IRRs, an exploratory objective of GREEN is to investigate alternative measures for obinutuzumab administration. A comparison of the IRR risk mitigation strategies employed in GREEN has been reported separately [41].

The inclusion criteria for patients in the G-FC subgroup included: aged ≥ 18 years with previously untreated CLL requiring treatment per the International Workshop on CLL criteria [4]; adequate hematologic function (defined as hemoglobin ≥ 9.0 g/dL, an absolute neutrophil count of ≥1.5 × 10^9^/L, and platelets ≥ 75 × 10^9^/L); and an Eastern Cooperative Oncology Group performance status of 0–2. All patients had to be fit (defined as a Cumulative Illness Rating Scale score of ≤6 and a creatinine clearance ≥70 mL/min) to receive FC as chemotherapy. Patients with a 17p deletion and/or *TP53* mutation could be included at the investigator’s discretion [41].

Patients received obinutuzumab 1000 mg intravenously (IV) on Day (D)1, 8, and 15 of Cycle (C)1, and on D1 of C2‒6 for six 28-day cycles. To explore potential measures to reduce the risk of IRRs, the C1D1 dose of obinutuzumab was administered over two days, and patients were allocated to three cohorts: 25 mg (12.5 mg/h; C1D1) + 975 mg (50–400 mg/h; C1D2) (Cohorts 1 and 3); 100 mg (25 mg/h; C1D1) + 900 mg (50–400 mg/h; C1D2) (Cohort 2). All patients received prednisolone IV 100 mg (or equivalent) 1 h pre dose on C1D1/D2, and patients in Cohorts 2 and 3 received additional corticosteroids (oral dexamethasone 20 mg or equivalent dose of betamethasone 16‒20 mg) 12 h before the first dose of obinutuzumab. FC were administered on D1–3 of each cycle at standard doses, either IV (fludarabine 25 mg/m^2^ over 30 min and cyclophosphamide 250 mg/m^2^ over 15–30 min) or orally (fludarabine 40 mg/m^2^ and cyclophosphamide 250 mg/m^2^).

Measures to minimize the risk of tumor lysis syndrome (TLS) in patients with high tumor burden (peripheral blood lymphocyte count ≥25 × 10^9^/L or bulky lymphadenopathy) were also applied; these included appropriate hydration, pretreatment with allopurinol or a suitable alternative, intensive laboratory monitoring, implementation of an expanded risk definition, investigator training, and patient education [40]. Use of granulocyte-colony stimulating factor was permitted, with primary prophylaxis recommended for patients aged ≥ 60 years and/or with comorbidities. Antimicrobial prophylaxis was not mandated by the study protocol in GREEN but could be given at the investigator’s discretion.

GREEN was conducted in accordance with the Declaration of Helsinki, Good Clinical Practice guidelines, and all applicable local laws and regulations. The study protocol and its amendments, and other study-related materials were approved by institutional review boards/ethics committees at participating centers. Written informed consent was provided by all patients.

### Study endpoints

The primary outcome measure was safety, including AEs, grade ≥ 3 AEs, serious AEs, AEs of special interest (AESI; IRRs, infections, neutropenia, and TLS), AEs of particular interest (AEPI; including thrombocytopenia, second malignancies, hepatitis B reactivation, and hemorrhagic events), dose delays/discontinuations, and laboratory abnormalities.

Secondary efficacy endpoints included overall response rate (ORR; defined as a confirmed complete response [CR] plus CR with incomplete marrow recovery [CRi], or partial response [PR], as determined by the study investigator, at the time of the final response assessment), duration of response (defined as the period from the date of initial confirmed PR or CR until the date of progressive disease or death from any cause), PFS (defined as the time from the date of treatment initiation until the first documented disease progression or death from any cause), time to new antileukemic therapy (defined as the time between the date of treatment initiation and first intake of new antileukemic therapy), and MRD negativity rate (defined as <1 CLL cell per 10,000 leukocytes) in peripheral blood or bone marrow.

### Assessments

AEs, serious AEs, and AESI/AEPI were monitored, and graded according to the National Cancer Institute’s Common Terminology Criteria for AEs version 4.0. IRRs were defined as any AE that occurred during or within 24 h of obinutuzumab infusion and were considered related to obinutuzumab. Other safety assessments included physical examinations, electrocardiogram, vital signs, and laboratory parameters.

Tumor response was assessed by investigators according to International Workshop on CLL criteria [4] at the final response assessment, which occurred ~3 months after the last dose of study treatment. A computed tomography scan was required to confirm CR and PR. A bone marrow biopsy was required for confirmation of CR. Patients lacking a valid biopsy, but who otherwise met CR criteria, were classed by the investigator as PR.

MRD was assessed in peripheral blood (all patients) or bone marrow (in patients requiring confirmation of CR), also at the final response assessment; analysis was by four-color flow cytometry, undertaken in a EuroFlow-certified central laboratory in Kiel, Germany [42, 43].

### Statistical methodology

There was no hypothesis testing in GREEN and therefore no power calculation. The sample size estimation of 950 patients for the overall study population was based on the ability to detect nonfrequent AEs (irrespective of grade) in order to provide insight into the overall safety profile of obinutuzumab alone or with chemotherapy. This is a prespecified subgroup analysis of fit patients treated with G-FC in the GREEN study.

The safety population comprised patients who received ≥1 dose of study treatment and was used for all safety analyses. Safety data are presented using descriptive statistics. Efficacy endpoints were assessed in all patients regardless of whether they received any therapy (intent-to-treat [ITT] population). For the MRD analyses, the intent-to-ship population comprised all patients whose MRD samples at the final response assessment could be shipped to the central laboratory within 48 h; centers unable to supply fresh samples to the central laboratory within 48 h of sampling were excluded from the MRD analyses. The MRD-evaluable population comprised all patients within the intent-to-ship population with an evaluable MRD result (peripheral blood or bone marrow).

The ORR, CR rate, and MRD negativity rate are presented with two-sided 95% Clopper–Pearson confidence intervals (CIs). Time-to-event endpoints were estimated using Kaplan–Meier methodology.

The analysis presented here formed part of the primary analysis and took place after all patients had undergone the final response assessment, which occurred ~3 months after completion of study treatment (data cutoff, December 29, 2016). After the data snapshot was taken for analysis, a few additional AEs (seven AEs in six patients) were reported late by sites on the database, which remained open to continue collecting information until the final analysis (Supplementary Table S1). In addition, one patient with CR at the final response assessment had this changed to CRi (Supplementary Table S2). These updates are not part of the statistical analysis or summary tables that are presented.

## Results

### Patients

Patients (including those reported in this subgroup analysis) were enrolled between October 2013 and March 2016 at 195 centers in 31 countries in Africa, North and South America, Asia, and Europe. Both the ITT and safety populations in this analysis comprised 140 fit patients with previously untreated CLL; all patients received G-FC treatment. At the time of analysis, 11 patients had discontinued the study, and 129 were still ongoing (all in follow-up). Primary reasons for study discontinuation were death (*n* = 4), withdrawal of consent (*n* = 4), AE (*n* = 1; IRR: cytokine release syndrome), investigator decision (*n* = 1), and other (*n* = 1).

Median age was 57 (range, 34‒74) years; 67.9% of patients were male, and most had advanced Binet stage at screening (Table 1). IGHV was mutated in 26.4% of patients, unmutated in 55.7%, and missing in 17.9%. Chromosomal abnormalities detected by fluorescence in situ hybridization were present in 75.7% of patients, 55.7% were ZAP70 positive, and 40.7% were CD38 positive.

**Table 1:** Baseline demographics and disease characteristics (G-FC population, previously untreated, fit)

Characteristic	All patients (*n* = 140)
Median age, years (range)	57 (34–74)
Male, *n* (%)	95 (67.9)
Median CIRS score (range)	2 (0–6)
ECOG performance status, *n* (%)	
0	108 (77.1)
1	32 (22.9)
Binet stage at screening, *n* (%)	
A	37 (26.4)
B	74 (52.9)
C	29 (20.7)
Absolute lymphocyte count, *n* (%) ≥50 × 10^9^/L	93 (66.4)
Tumor bulk ≥5 cm, *n* (%)	101 (72.1)
Genomic aberrations, *n* (%)*	
17p deletion	4 (2.9)
11q deletion	30 (21.4)
12q trisomy	20 (14.3)
13q deletion	43 (30.7)
Other aberrations	9 (6.4)
No abnormality	18 (12.9)
Missing	16 (11.4)
IGHV, *n* (%)	
Unmutated	78 (55.7)
Mutated	37 (26.4)
Missing	25 (17.9)
ZAP70, *n* (%)	
Positive	78 (55.7)
Negative	40 (28.6)
Missing^†^	22 (15.7)
CD38, *n* (%)	
Positive	57 (40.7)
Negative	60 (42.9)
Missing^‡^	23 (16.4)

*CIRS* Cumulative Illness Rating Scale, *CrCl* creatinine clearance, *ECOG* Eastern Cooperative Oncology Group, *G-FC* obinutuzumab plus fludarabine and cyclophosphamide, *IGHV* immunoglobulin heavy chain variable region

*According to the hierarchical model of genomic aberrations

†22 patients had missing central lab evaluation, of whom 6 had local lab assessment (3 positive, 3 negative) and the remaining 16 patients had missing local lab evaluation

^‡^7 patients had local lab assessment (5 positive, 2 negative)

### Treatment exposure

**Fig. 1:**
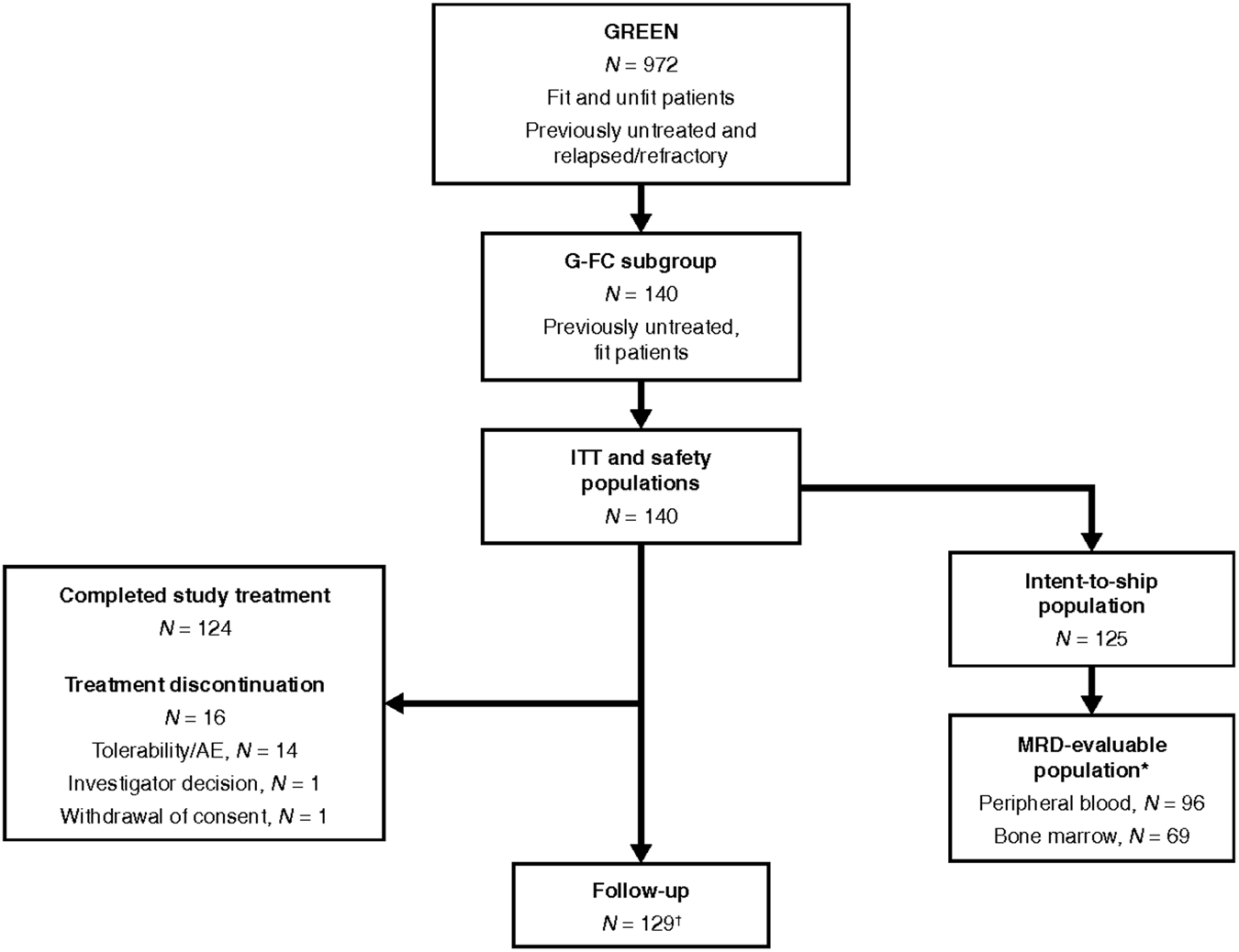
Patient flow diagram. *Comprised all patients within the intent-to-ship population with an evaluable MRD result (peripheral blood or bone marrow); ^†^still ongoing. AE adverse event, G-FC obinutuzumab plus fludarabine and cyclophosphamide, ITT intent-to-treat, MRD minimal residual disease

In total, 124 patients (88.6%) completed all study treatment per protocol. Reasons for not completing study treatment were tolerability/AEs (*n* = 14), investigator’s decision (*n* = 1), and withdrawal of consent (*n* = 1) (Fig. 1).

Mean number of obinutuzumab administrations was 8.8 (planned, 9), with 95.0% (*n* = 133) of patients receiving ≥90% of the planned dose. Median obinutuzumab exposure time was 21.1 (range, 0.3‒28.1) weeks. Mean number of chemotherapy cycles administered was 5.7 for both cyclophosphamide and fludarabine (planned, 6), with 94.3% of patients (*n* = 132) receiving ≥90% of the planned doses of both drugs. In total, 104 patients (74.3%) received previous or concomitant granulocyte-colony stimulating factor administration.

### Safety

Median observation time was 25.6 (range, 2.0–37.8) months. In the safety analysis, most patients (98.6%; *n* = 138) reported ≥1 AE of any grade. The most frequently reported treatment-emergent AEs of any grade (by preferred term, affecting ≥20% of patients) were neutropenia (75.7%), nausea (42.1%), pyrexia (37.1%), thrombocytopenia (35.0%), anemia (25.7%), vomiting (25.7%), and diarrhea (20.7%). AEs were considered related to treatment in 95.7% of patients, most commonly neutropenia (72.9%), nausea (38.6%), thrombocytopenia (32.9%), pyrexia (28.6%), vomiting (21.4%), and anemia (20.7%).

In total, 87.1% of patients experienced grade ≥ 3 AEs (Table 2), the most common of which (affecting >5% of patients) were neutropenia (67.1%), thrombocytopenia (17.1%), anemia (10.7%), leukopenia (7.9%), febrile neutropenia (7.1%), and pneumonia (5.7%). Serious AEs were experienced by 42.1% of patients (Table 2), and included neutropenia (15.7%), febrile neutropenia (6.4%), pneumonia (5.7%), and pyrexia (5.7%).

**Table 2:** Safety overview (safety population)

	Number (%) of patients reporting AEs (*n* = 140)
Grade ≥ 3 AEs by preferred term (reported by ≥2% of patients)	
Any	122 (87.1)
Neutropenia	94 (67.1)
Thrombocytopenia	24 (17.1)
Anemia	15 (10.7)
Leukopenia	11 (7.9)
Febrile neutropenia	10 (7.1)
Pneumonia	8 (5.7)
Lymphopenia	7 (5.0)
Hypertension	4 (2.9)
Hyperglycemia	4 (2.9)
Neutrophil count decreased	4 (2.9)
TLS	3 (2.1)
Hypotension	3 (2.1)
Lung infection	3 (2.1)
Pyrexia	3 (2.1)
SAEs by preferred term (reported by ≥2% of patients)	
Any	59 (42.1)
Neutropenia	22 (15.7)
Febrile neutropenia	9 (6.4)
Pneumonia	8 (5.7)
Pyrexia	8 (5.7)
Thrombocytopenia	4 (2.9)
Anemia	3 (2.1)
Lung infection	3 (2.1)
Grade ≥ 3 AESI/AEPI (basket terms)	
Neutropenia*	99 (70.7)
IRRs^†^	27 (19.3)
Thrombocytopenia^‡^	25 (17.9)
Infections^§^	22 (15.7)
Second malignancies by MedDRA SOC^¶^	5 (3.6)
TLS**	3 (2.1)
Hepatitis B reactivation^††^	0
Hemorrhagic events^‡‡^	0

*AEs* adverse events, *AEPI* adverse events of particular interest, *AESI* adverse events of special interest, *IRR* infusion-related reaction, *MedDRA* Medical Dictionary for Regulatory Activities, *SAE* serious adverse event, *SMQ* standardized MedDRA queries, *SOC* system organ class, *TLS* tumor lysis syndrome

*Includes neutropenia, febrile neutropenia, neutrophil count decreased, and neutropenic sepsis

^†^AEs that occurred during or within 24 h of obinutuzumab infusion and considered related to obinutuzumab

^‡^Includes thrombocytopenia and platelet count decreased

^§^All AEs classified as infections and infestations (MedDRA SOC)

^¶^Second malignancy including benign, malignant, and unspecified tumors (MedDRA SOC) occurring > 6 months after first study drug intake

**Any AE with the preferred term “TLS”; one patient was classified as having both TLS and an IRR

^††^Any AE with the preferred term containing “hepatitis B” or “hepatitis acute” that was additionally assessed as hepatitis B virus reactivation via medical review

^‡‡^Includes hematoma, epistaxis, hematuria, conjunctival hemorrhage, ecchymosis, hematochezia, hematospermia, and metrorrhagia

AESI/AEPI (basket terms) included neutropenia (any grade, 77.9%, grade ≥ 3, 70.7%), IRRs (any grade, 69.3%; grade ≥ 3, 19.3% [Supplementary Table S3]), infections (any grade, 56.4%, grade ≥ 3, 15.7%), thrombocytopenia (any grade, 37.1%, grade ≥3, 17.9%), second malignancies according to Medical Dictionary for Regulatory Activities (MedDRA) system organ class (any grade, 4.3%; grade ≥ 3, 3.6%), and TLS (any grade, 2.1%; all grade 3, and all laboratory TLS) (Table 2). Sixty patients (55.0%) required a modified treatment regimen (obinutuzumab or FC) due to neutropenia (reported as an AESI/AEPI). The most common infections (affecting ≥5% of patients) by preferred term were bronchitis (8.6%), pneumonia (7.1%), nasopharyngitis (7.1%), upper respiratory tract infection (6.4%), influenza (5.7%), sinusitis (5.7%), and herpes zoster (5.0%). The majority of infections were bacterial; there were no cases of pneumocystis pneumonia infection, and one case each of candida and fungal infection. Opportunistic infections included two cases of herpes simplex virus infection, and one case each of varicella zoster virus, listeriosis, and lymph node tuberculosis infection.

Fourteen patients (10.0%) discontinued G-FC treatment prematurely because of AEs, most common AESI/AEPI were neutropenia (3.6%; *n* = 5), thrombocytopenia (2.1%; *n* = 3), or infections (1.4%; *n* = 2). There were four AEs leading to death (one case each of sepsis, second malignancy [acute myeloid leukemia], pneumonia, and unexplained death) (Supplementary Table S4); three of the four patients who died had previously discontinued treatment due to AEs. One additional patient died due to PD.

### Response rate at final response assessment

ORR at the final response assessment was 90.0% (95% CI: 83.8‒94.4), with 46.4% of patients achieving a CR (including CRi) and 43.6% of patients achieving a PR (Table 3). Response was missing or not evaluable in eleven patients (7.9%). Of the four patients with 17p deletions, one achieved CR and one had progressive disease (response not available in two patients).

**Table 3:** Response rates at the final response assessment (investigator assessment; ITT population)

*n* (%) [95% CI]	All patients (*n* = 140)
ORR	126 (90.0) [83.8–94.4]
CR	37 (26.4) [19.3–34.5]
CRi	28 (20.0) [13.7–27.6]
PR	61 (43.6) [35.2–52.2]
SD	1 (0.7) [0.0–3.9]
PD	2 (1.4) [0.2–5.1]
Missing or not evaluable	11 (7.9)

*CI* confidence interval, *CR* complete response, *CRi* complete response with incomplete marrow recovery, *ITT* intent-to-treat, *ORR* overall response rate, *PD* progressive disease, *PR* partial response, *SD* stable disease

### MRD negativity rate at final response assessment

For the ITT population, MRD negativity rates were 64.3% (90/140) and 35.7% (50/140) in peripheral blood and bone marrow, respectively (Table 4). MRD negativity rates in the intent-to-ship population, comprising 125 patients, were 72.0% (90/125) in peripheral blood and 40.0% (50/125) in bone marrow. The MRD-evaluable population comprised 96 patients with an evaluable peripheral blood sample and 69 patients with a bone marrow sample. MRD negativity rates in the MRD-evaluable population were 93.8% (90/96) and 72.5% (50/69) for peripheral blood and bone marrow, respectively.

**Table 4:** MRD negativity rates and overall response in MRD-negative patients at the final response assessment

*n/N* (%)	All patients (*n* = 140)
Peripheral blood	
Intent-to-treat population	*n* = *140*
MRD negative	90/140 (64.3)
Intent-to-ship population	*n* = *125*
MRD negative	90/125 (72.0)
Evaluable population	*n* = *96*
MRD negative	90/96 (93.8)
CR	26/90 (28.9)
CRi	19/90 (21.1)
PR	41/90 (45.6)
SD	1/90 (1.1)
Missing	3
Bone marrow^a^	
Intent-to-treat population	*n* = *140*
MRD negative	50/140 (35.7)
Intent-to-ship population	*n* = *125*
MRD negative	50/125 (40.0)
Evaluable population	*n* = *69*
MRD negative	50/69 (72.5)

*CR* complete response, *CRi* complete response with incomplete marrow recovery, *G-FC* obinutuzumab plus fludarabine and cyclophosphamide, *MRD* minimal residual disease, *PR* partial response, *SD* stable disease

^a^Bone marrow samples were only collected from patients with suspected CR or CRi at the final response assessment

Among IGHV mutated patients (*n* = 37) with an evaluable peripheral blood (*n* = 29) and bone marrow sample (*n* = 21), MRD negativity rates were 96.6% (28/29) and 66.7% (14/21), respectively. In IGHV unmutated patients (*n* = 77), MRD negativity rates were 91.5% (54/59) and 72.1% (31/43) in peripheral blood and bone marrow, respectively (Supplementary Table S5).

### Progression-free survival and time to new antileukemic therapy

**Fig. 2:**
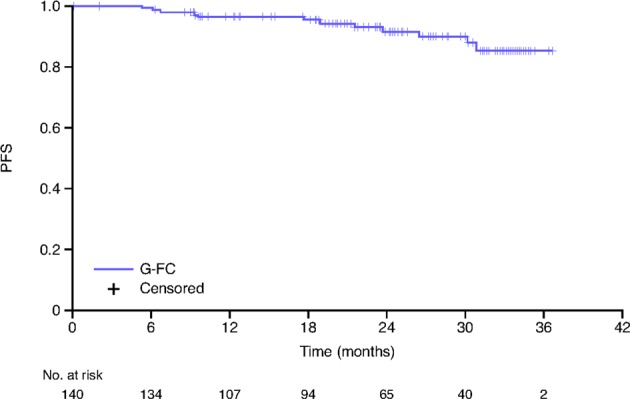
Kaplan−Meier plot of progression-free survival (ITT population). G-FC obinutuzumab plus fludarabine and cyclophosphamide, ITT intent-to-treat, PFS progression-free survival

After a median observation time of 25.6 (range, 2.0–37.8) months and 12 PFS events, median PFS was not reached (Fig. 2). At 2 years, estimated PFS was 91% (95% CI: 84‒96%). Among IGHV mutated (*n* = 37) and unmutated (*n* = 78) patients, 2 year PFS was 96% (95% CI: 75‒99%) and 88% (95% CI: 76‒94%), respectively (Supplementary Fig. S1). Six patients received new antileukemia therapy, with time to new antileukemic therapy in these patients ranging from 1.2 to 37.8 months.

A meaningful analysis of PFS according to MRD response was not possible because of the small number MRD-positive patients (*n* = 5 in peripheral blood) and bias introduced by withdrawn patients not undergoing MRD evaluation at the final response assessment.

## Discussion

The safety and efficacy of frontline G-FC was assessed in 140 fit CLL patients. Results from this subgroup analysis of the international, phase 3b GREEN study largely support the preliminary findings of the phase 1b GALTON study [39], and suggest that G-FC may have manageable toxicity and promising efficacy in this patient population. Strengths and limitations of the GREEN study have been described previously [41].

AEs reported in this study were clinically relevant but were consistent with the known safety profile of obinutuzumab [14, 17, 18, 37–39], with the most common being hematologic AEs, IRRs, and infections. The high rate of some AEs, particularly hematologic events, reflects the intensity of this treatment regimen; however, most patients could tolerate treatment, with only 10.0% discontinuing G-FC due to AEs. No unexpected safety signals were observed, although the occurrence of IRRs, including TLS, highlights the need for careful risk assessment, prophylaxis, and monitoring of these events.

The rate of grade ≥3 neutropenia in this analysis was 67.1%. Among fit patients receiving frontline R-FC, incidence of grade ≥3 neutropenia was 84.2% in the CLL10 study [12] and 34% (grade 3‒4) in the CLL8 study [20]. Some of the differences between studies can be accounted for by differences in safety observation times. Importantly, the relatively high rate of grade ≥3 neutropenia (70.7%) in the current analysis did not translate into a high rate of infection (15.7%) [20]. The percentage of patients who experienced grade ≥3 neutropenia was higher than that seen with the combination of G-B (48.6% [fit patients only]) in the GREEN study [40], which is in accordance with the finding that R-B is associated with a lower incidence of neutropenia than R-FC, as reported in CLL10 [12].

Grade ≥3 IRRs were experienced in 19.3% of patients, and most occurred during the first infusion. Of note, this rate of grade ≥3 IRRs is higher than that generally seen for patients receiving rituximab-based chemoimmunotherapy; this may be owing to the greater level of B-cell depletion and accompanying proinflammatory cytokine release achieved with obinutuzumab compared with rituximab [44]. In other reports of obinutuzumab-based chemoimmunotherapy, grade ≥3 IRRs were observed in 20% of patients treated with G-Clb in CLL11 [14] and in 17.1% of patients receiving G-B in Cohort 1 of GREEN [40]. No IRRs were fatal in this analysis.

In total, four patients died (2.9%) due to an AE and 1 (0.7%) patient died due to progression of disease. In comparison, incidence of treatment-related deaths with R-FC was 2.0% in CLL8 [20] and 5% in CLL10 [12].

ORR (90.0%), CR rate (46.4%, including CRi), and PFS rate (91% at 2 years) were favorable, suggesting that G-FC is clinically active in this setting. These findings are consistent with other studies, which have demonstrated the effectiveness of frontline immunochemotherapy in CLL, particularly in patients with a good biologic profile [11–13, 20–23, 40]. A high rate of MRD negativity was also achieved with G-FC (64.3% in peripheral blood [72.0% of the intent-to-ship and 93.8% of the MRD-evaluable population] and 35.7% in bone marrow [40.0% of the intent-to-ship and 72.5% of the MRD-evaluable population]), which is particularly encouraging given the increasing evidence linking posttreatment MRD status with therapeutic outcome [24–31]. The discrepancy between the CR and MRD negativity rates in the present study is, however, currently unclear. In CLL10, the MRD negativity rate reported for fit CLL patients receiving frontline R-FC was 74% in peripheral blood and 58% in bone marrow (in the MRD-evaluable population only) [12]. Interestingly, the level of MRD negativity achieved with G-FC is only slightly higher than that achieved with G-B in the GREEN study (despite unfit and fit patients receiving G-B), although meaningful comparisons between regimens cannot be made due to the nonrandomized allocation of chemotherapy (a limitation of the study) and differences in sample size [40].

Nowadays, MRD can reliably be detected to a level of one CLL cell in 10,000 leukocytes. Although not routinely performed in clinical practice, MRD assessment has been included in many clinical trials using the flow cytometry technique harmonized and validated by the European Research Initiative on CLL [35, 38, 39]. Several of these trials have reported that MRD negativity is independently associated with longer PFS and OS in CLL patients after frontline therapy [24–31]. Recently, MRD has been identified as a potential surrogate marker for PFS [45], suggesting it could provide an early indication of efficacy. Such findings highlight the importance of utilizing MRD-negative remission as a standard endpoint in clinical trials.

Estimated 2 year PFS rates were higher for IGHV mutated patients (96%) treated with G-FC compared with IGHV unmutated patients (88%), while MRD negativity rates were comparable. This latter result appears to show high MRD response rates with G-FC regardless of IGHV mutation status, but this may also be partly due to biasing the results of MRD in peripheral blood within the intent-to-ship population (*n* = 125), as only a subset of these patients were MRD-evaluable (*n* = 96). Results from three separate studies suggest that CLL patients with mutated IGHV benefit substantially from frontline R-FC chemoimmunotherapy, and may even be cured with this regimen [11, 23, 35].

Baseline characteristics were generally consistent with “typical” frontline, fit patients with CLL. The incidence of 17p deletions (2.9%) was low (typically 5–8%) [36], likely reflecting an increasing move towards risk-adapted therapy. Patients with 17p deletions show a marked resistance to chemotherapy, which cannot be overcome by the addition of anti-CD20 antibodies [2, 20]. Instead, these patients should ideally be treated with novel agents like ibrutinib, unless contraindications suggest otherwise [2].

Ibrutinib has emerged as a well-tolerated and highly effective treatment option for CLL patients in the relapse setting, and there is a growing consensus in favor of frontline therapy with ibrutinib in elderly CLL patients and in CLL patients with poor prognostic factors [6–8]. It also has potential to be used in combination with rituximab and/or immunochemotherapy in fit patients [32]. However, while ibrutinib may be associated with good outcomes and a favorable toxicity profile [6], long-term AEs and the impact of its cost on the health care systems remain to be elucidated [46, 47]. There is also a need to better understand the reasons behind the high discontinuation rates reported for ibrutinib in real-life studies [48, 49].

In conclusion, G-FC represents a promising treatment option for young, fit patients with previously untreated CLL who are eligible for potent chemoimmunotherapy. Moreover, G-FC could hold potential as an effective “backbone” of combination therapies with new compounds, such as ibrutinib; such a regimen (G-FC plus ibrutinib) is already showing favorable efficacy in a phase 2 trial as first-line treatment for fit CLL patients with mutated IGHV [50]. No unexpected safety signals were observed with G-FC in the present study and side effects were generally manageable, although the high occurrence of neutropenia and IRRs, including TLS, highlights the need for careful risk assessment, prophylaxis, and monitoring of these events. The low rate of progression and high rate of MRD negativity indicate that G-FC is a clinically active treatment option for fit patients with CLL, although longer follow-up is required to confirm these results.

## Supplementary information

Supplementary figure 1

Supplementary material

## Data Availability

“Qualified researchers may request access to individual patient level data through the clinical study data request platform (www.clinicalstudydatarequest.com). Further details on Roche’s criteria for eligible studies are available here (https://clinicalstudydatarequest.com/Study-Sponsors/Study-Sponsors-Roche.aspx). For further details on Roche’s Global Policy on the Sharing of Clinical Information and how to request access to related clinical study documents, see here (https://www.roche.com/research_and_development/who_we_are_how_we_work/clinical_trials/our_commitment_to_data_sharing.htm)”
