# Supplementary figures and images for: Obinutuzumab plus fludarabine and cyclophosphamide in previously untreated, fit patients with chronic lymphocytic leukemia: a subgroup analysis of the GREEN study
Source: Leukemia. 2019 Aug 27;34(2):441–50. doi: 10.1038/s41375-019-0554-1 (PMC7214269; doi:10.1038/s41375-019-0554-1)

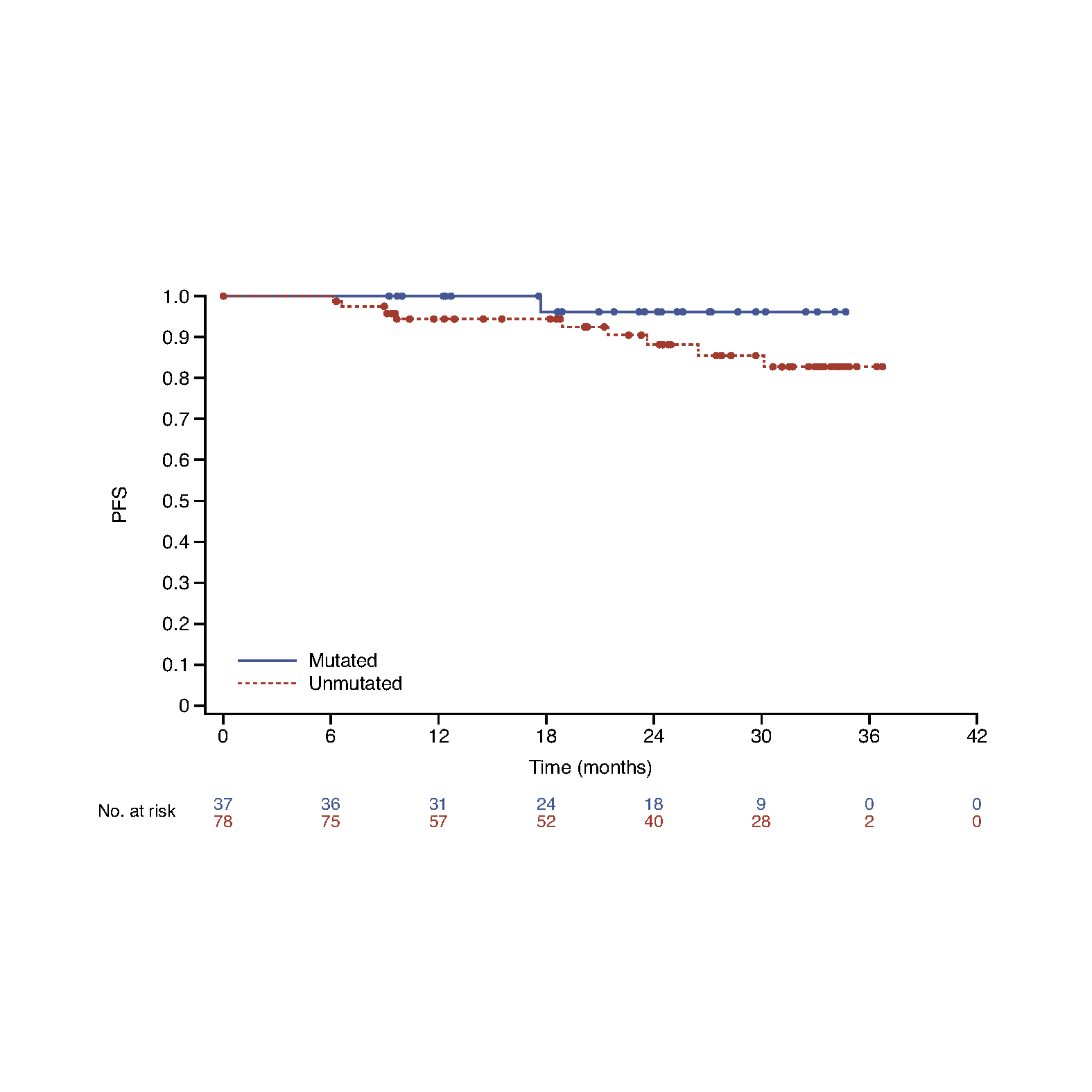

Supplement: Supplementary file 1 — Supplementary figure 1 [file 41375_2019_554_MOESM1_ESM.tif]
