# Supplementary material for: Obinutuzumab plus fludarabine and cyclophosphamide in previously untreated, fit patients with chronic lymphocytic leukemia: a subgroup analysis of the GREEN study
Source: Leukemia. 2019 Aug 27;34(2):441–50. doi: 10.1038/s41375-019-0554-1 (PMC7214269; doi:10.1038/s41375-019-0554-1)
Supplement: Supplementary file 2 — Supplementary material [file 41375_2019_554_MOESM2_ESM.docx]

**Supplementary information**

The supplementary information includes five supplementary tables and one supplementary figure.

**Table S1.** Adverse events not considered in the manuscript that occurred prior to cut-off but were reported after the data
analysis snapshot

| AE | Age, years  (gender) | Onset  (study day) | End  (study day) | Grade | SAE | Relationship to obinutuzumab/ chemotherapy | Outcome |
| --- | --- | --- | --- | --- | --- | --- | --- |
| Hepatitis E | 58 (F) | 570 | 1024 | 3 | Y | Related to obinutuzumab and chemotherapy | Resolved |
| Neutropenia | 66 (M) | 113 | NA | 2 | N | Related to chemotherapy | Ongoing |
| Myelosuppression | 61 (M) | 181 | 203 | 3 | N | Related to chemotherapy | Resolved |
| Headache | 41 (M) | NA | 755 | 3 | Y | Not related | Resolved with sequelae |
| Fatigue* | 58 (F) | 161 | NA | 1 | N | Related to obinutuzumab and chemotherapy | Ongoing |
| Neuropathy* | 58 (F) | 245 | 480 | 1 | N | Related to obinutuzumab and chemotherapy | Resolved |
| Febrile neutropenia | 50 (M) | 99 | 99 | 3 | Y | Related to obinutuzumab and chemotherapy | Resolved |

*AEs occurred in the same patient. AE, adverse event; F, female; M, male; N, no; NA, not available; SAE, serious adverse event; Y, yes.

**Table S2.** Change in final response assessment*

|  | Age, years (gender) | Primary analysis snapshot | Updated to |
| --- | --- | --- | --- |
| Response | 58 (M) | CR | CRi |

*****After the data analysis snapshot was taken, one response was changed from CR to CRi by a site on the database that remained open to continue collecting information until the final analysis.

CR, complete response; CRi, complete response with incomplete marrow recovery; M, male.

**Table S3.** Summary of infusion-related reactions (safety population)

|  | *N* (%) of patients reporting IRRs  (*N* = 140) |
| --- | --- |
| Any IRR | 97 (69.3) |
| Grade ≥3 IRRs | 27 (19.3) |
| Serious IRRs | 13 (9.3) |
| IRRs leading to treatment discontinuation | 1 (<1.0) |
| IRRs (reported by ≥2% patients, any grade by preferred term) |  |
| Pyrexia | 27 (19.3) |
| Nausea | 21 (15.0) |
| Chills | 11 (7.9) |
| Vomiting | 10 (7.1) |
| Dyspnea | 10 (7.1) |
| Rash | 9 (6.4) |
| Hypertension | 8 (5.7) |
| Hyperhidrosis | 7 (5.0) |
| Chest discomfort | 7 (5.0) |
| Hyperthermia | 7 (5.0) |
| Hypotension | 7 (5.0) |
| Headache | 6 (4.3) |
| Thrombocytopenia | 6 (4.3) |
| Alanine aminotransferase increased | 5 (3.6) |
| Aspartate aminotransferase increased | 5 (3.6) |
| Hot flush | 5 (3.6) |
| Cytokine release syndrome | 4 (2.9) |
| Tachycardia | 4 (2.9) |
| Asthenia | 3 (2.1) |
| Anemia | 3 (2.1) |
| Neutropenia | 3 (2.1) |
| Tumor lysis syndrome | 3 (2.1) |

IRRs, infusion-related reactions.

**Table S4.** Listing of grade 5 (fatal) adverse events (safety population)

| AE (preferred term) | Age, years (gender) | Treatment period (onset of event)* | AESI | AEPI | Relationship to obinutuzumab/ chemotherapy |
| --- | --- | --- | --- | --- | --- |
| Unexplained death | 65 (M) | Post (Day 146) | N | N | Not related |
| Sepsis | 74 (F) | Post (Day 180) | Y | N | Not related |
| Acute fibrinous organizing pneumonia | 47 (F) | During (Day 105) | N | N | Related to obinutuzumab and chemotherapy |
| Acute myeloid leukemia | 64 (M) | Post (Day 903) | N | Y | Not related |

*****Post = post treatment period; during = during treatment period.

AE, adverse event; AEPI, adverse events of particular interest; AESI, adverse event of special interest; F, female; M, male; N, no; Y, yes.

**Table S5.** Minimal residual disease response in peripheral blood and bone marrow according to IGHV mutation status

| n/N (%) | All patients (*N* = 140) |
| --- | --- |
| **Peripheral blood** | |
| IGHV mutated | *N* = 37 |
| *Number of patients included in analysis* | 29 |
| MRD negative | 28/29 (96.6) |
| MRD positive | 0 |
| MRD unknown | 1/29 (3.4) |
| IGHV unmutated | *N* = 77 |
| *Number of patients included in analysis* | 59 |
| MRD negative | 54/59 (91.5) |
| MRD positive | 5/59 (8.5) |
| **Bone marrow** | |
| IGHV mutated | *N* = 37 |
| *Number of patients included in analysis* | 21 |
| MRD negative | 14/21 (66.7) |
| MRD positive | 7/21 (33.3) |
| IGHV unmutated | *N* = 77 |
| *Number of patients included in analysis* | 43 |
| MRD negative | 31/43 (72.1) |
| MRD positive | 11/43 (25.6) |
| MRD unknown | 1 (2.3) |

IGHV, immunoglobulin heavy chain variable region; MRD, minimal residual disease.

**Fig. S1.** Kaplan−Meier plot of progression-free survival according to IGHV mutation status (ITT population)


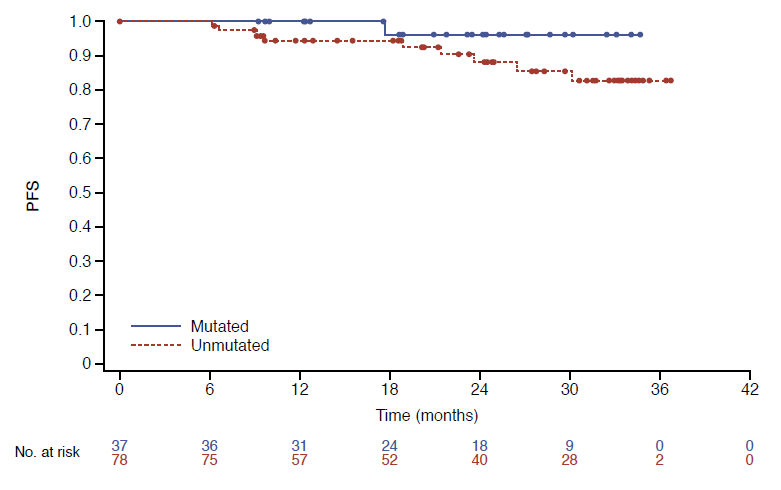


IGHV, immunoglobulin heavy chain variable region; ITT, intent-to-treat; PFS, progression-free survival.
